# Identifying non-responders to a multifactorial intervention to improve glycemic control in elderly diabetic patients

**DOI:** 10.1186/1758-5996-7-S1-A52

**Published:** 2015-11-11

**Authors:** Rafael Vaz Machry, Henrique Umpierre Pedroso, Rafaela Ramos Nunes, Cibelle de Abreu Evaldt, Luthiele da Silva Vasconcellos, Thaymê Luísa de Souza Pires, Raquel Ferreira, Eduardo Bardou Yunes Filho, Paloma Dias da Cruz, Ticiana da Costa Rodrigues

**Affiliations:** 1 UFRGS, Porto Alegre, Brazil

## Background

There are some strategies to improve adherence (AD) and glycemic control (GC). Some barriers may be faced by elderly diabetics. To identify factors that hinder the treatment is essential.

## Objectives

To evaluate the glycemic response after changing the syringes (SY) for pens device (PD), and to identify possible “non-responders” (NR) to this treatment.

## Materials and methods

This is a prospective, intervention, non-randomized, phase IV study. We included patients over 60 yrs. old, both sexes, with HbA1c>8.5% using oral hypoglycemic agents and insulin (IN), then we replaced SY by PD. IN used were NPH and Regular, all patients have received a blood glucose monitor, lancet tapes, capillary blood glucose tests (3 tests/day). HbA1c was measured at baseline, 3 and 6 months. Patients were seen monthly. We considered “responders” (R) who showed improvement in HbA1c of more than 0.4% after first 3 months (as used on clinical practice).

## Results

38 patients completed follow-up in the first quarter. 31 were considered R and 7 were considered NR. There were no differences between the two groups for age, gender, education, family income, race, religion, history of smoking and alcohol consumption or presence of chronic complications. R showed greater variation (delta) of HbA1c in the first trimester compared to NR (-2.17±1.33 vs. +0.35±0.99, p<0.001), and in six months (R -2.40±1.36 vs. NR - 0.91±1.26, p=0.013). There was no difference in the change in the second trimester, (R - 0.27±1.02 vs. NR 1.99±-1.27, p=0.238) and in HbA1c – at baseline (R 10.28±1.5 vs. NR 9.78±1.88) and final (R 7.85±1.15 vs. NR 8.87±1.5), except in three months (R 8.10±1.09 vs. NR 10.14±1.88, p<0.001). There were no differences in the use of regular, number of daily injections, total daily IN dose (UI/kg) or in relation to AD – used IN doses counting (p=0.62). R had higher rates of sulfonylurea use associated with metformin and IN (p=0.001), and showed higher incidence of hypoglycemia (p=0.009) until the sixth visit, with no differences in severity and presence of nocturnal or asymptomatic hypoglycemia.

## Conclusion

We have found no differences in socio-cultural characteristics or AD rates to justify different on response. R had higher incidence of hypoglycemia and the use of IN associated with sulfonylureas can increase this tendency. At final, both groups had similar GC, and at this time, hypoglycemia was not different between them. Grants from CNPq and Fundo de Incentivo a Pq. do HCPA (FIPE).

